# Supporting Electrolyte Manipulation for Simple Improvement of the Sensitivity of Trace Vanadium(V) Determination at a Lead-Coated Glassy Carbon Electrode

**DOI:** 10.3390/s22218209

**Published:** 2022-10-26

**Authors:** Katarzyna Tyszczuk-Rotko, Damian Gorylewski, Jędrzej Kozak

**Affiliations:** Institute of Chemical Sciences, Faculty of Chemistry, Maria Curie-Skłodowska University, 20-031 Lublin, Poland

**Keywords:** vanadium, lead-coated glassy carbon electrode, adsorptive stripping voltammetry, supporting electrolyte manipulation, sensitivity improvement

## Abstract

The paper presents a very simple way to extremely improve the sensitivity of trace V(V) determination. The application of a new supporting electrolyte composition (CH_3_COONH_4_, CH_3_COOH, and NH_4_Cl) instead of the commonly used acetate buffer (CH_3_COONa and CH_3_COOH) significantly enhanced the adsorptive stripping voltammetric signal of vanadium(V) at the lead-coated glassy carbon electrode (GCE/PbF). A higher enhancement was attained in the presence of cupferron as a complexing agent (approximately 10 times V(V) signal amplification) than in the case of chloranilic acid and bromate ions (approximately 0.5 times V(V) signal amplification). Therefore, the adsorptive stripping voltammetric system with the accumulation of V(V)–cupferron complexes at −1.1 V for 15 s in the buffer solution (CH_3_COONH_4_, CH_3_COOH, and NH_4_Cl) of pH = 5.6 ± 0.1 was selected for the development of a simple and extremely sensitive V(V) analysis procedure. Under optimized conditions, the sensitivity of the procedure was 6.30 µA/nmol L^−1^. The cathodic peak current of V(V) was directly proportional to its concentration in the ranges of 1.0 × 10^−11^ to 2.0 × 10^−10^ mol L^−1^ and 2.0 × 10^−10^ to 1.0 × 10^−8^ mol L^−1^. Among the electrochemical procedures, the lowest detection limit (2.8 × 10^−12^ mol L^−1^) of V(V) was obtained for the shortest accumulation time (15 s). The high accuracy of the procedure was confirmed on the basis of the analysis of certified reference material (estuarine water) and river water samples.

## 1. Introduction

Vanadium is widespread in the earth’s crust but in low abundance. It is widely used and released in a wide variety of industrial processes. Its trace amount is essential for normal cell growth but can be toxic if present at higher concentrations. Vanadium(V), which occurs as VO_2_^+^ in an acidic solution and VO_4_^3−^ in an alkaline solution, is expected to be the predominant form in waters exposed to atmospheric oxygen [1]. The concentration of vanadium(V) in natural waters ranges from 10^−9^ and 10^−7^ mol L^−1^ [2,3] and, therefore, powerful analytical techniques are required for vanadium analysis. Just a few techniques, such as high-performance liquid chromatography, calibration-free laser-induced breakdown spectroscopy, electrothermal atomic absorption spectrometry, neutron activation analysis, inductively coupled plasma atomic spectrometry, mass spectrometry, and stripping voltammetry (SV), can meet the challenge of vanadium trace analysis in environmental water samples [4,5,6,7,8,9,10,11]. Of these techniques, only the SV can be used in routine field vanadium analysis. Adsorptive stripping voltammetry (AdSV), based on the accumulation of vanadium complexes with various complexing agents (such as chloranilic acid [11,12,13,14], cupferron [15,16], gallic acid [17], alizarin red S [18], alizarin violet [19], 2,3-dihydrobenzaldehide [20], and quercetin-5-sufonic acid [21]) on the electrode surface, has proven to be especially useful for the trace determination of vanadium. As can be seen, the most commonly used complexing agents are chloranilic acid and cupferron. Moreover, several voltammetric analytical procedures involve catalytic improvement of V(V) analytical signal due to the use of bromate ions [12,13,14,17,20]. To the best of our knowledge, the lowest detection limit of 4.5 × 10^−12^ mol L^−1^ for the accumulation time of 30 s was achieved at the hanging mercury drop electrode (HMDE) [21]. A limitation of that analytical procedure, particularly for field analysis, is the application of a stationary HMDE. Moreover, the toxicity of mercury limits the use of mercury electrodes in the analytical practice. Less toxic electrodes, such as bismuth-based electrodes, were applied to V(V) determination [12,16,17]. However, the detection limit of bismuth-based electrodes (2.5 × 10^−10^ mol L^−1^) is higher than that of HMDE. Therefore, an alternative to mercury-drop and the less toxic already-described electrodes that maintains the required sensitivity for trace V(V) determination is highly desired.

In our earlier works, we showed a simple way to amplify the analytical signal of U(VI) and Mo(VI) using lead film modified screen-printed carbon electrodes and a new supporting electrolyte composition (CH_3_COONH_4_, CH_3_COOH, and NH_4_Cl) [22,23]. The lead-coated electrode exhibited interesting characteristics, such as a wide potential window and the ability to operate in a wide range of pH media, lower toxicity and volatility compared with the mercury electrodes, good reproducibility, simple preparation, and a simple way of electrochemical surface renewal [24]. These advantages of the lead-coated electrode were already used for the V(V) analysis by AdSV [15]. However, in that procedure the acetate buffer solution of a pH equal to 5.6 was applied as the supporting electrolyte. It allowed it to reach the detection limit of 3.2 × 10^−10^ mol L^−1^ for the accumulation time of V(V)–cupferron complexes of 30 s, which is two orders of magnitude higher than that of HMDE. Therefore, the purpose of the present study was to evaluate the electrochemical properties of the adsorptive stripping system employing V(V)-chloranilic acid-BrO_3_^−^, V(V)–cupferron, and a new supporting electrolyte based on CH_3_COONH_4_, CH_3_COOH, and NH_4_Cl, as well as to develop a simple and extremely sensitive voltammetric procedure of V(V) determination using a lead-coated glassy carbon electrode (GCE/PbF).

## 2. Materials and Methods

### 2.1. Reagents

The supporting electrolyte (buffer solution of CH_3_COONH_4_, CH_3_COOH, and NH_4_Cl of pH = 5.6 ± 0.1) was prepared by mixing 1 mol L^−1^ CH_3_COONH_4_ (from 5 mol L^−1^ CH_3_COONH_4_, Sigma-Aldrich, Saint Louis, MO, USA) with a certain volume of 1 mol L^−1^ HCl (from 30% reagent of Fluka, Charlotte, NC, USA). The vanadium standard for ICP of 1 g L^−1^, cupferron (*N*-nitroso-*N*-phenylhydroxylamineammonium salt), chloranilic acid (2,5-dichloro-3,6-dihydroxy-*p*-benzoquinone), lead(II) nitrate, and potassium bromate were bought from Sigma-Aldrich (Saint Louis, MO, USA). The V(V) solutions of 1.0 × 10^−4^ and 1.0 × 10^−6^ mol L^−1^ were prepared in 1.0 × 10^−2^ mol L^−1^ NaOH. The solutions of 2.0 × 10^−2^ mol L^−1^ cupferron, 1.0 × 10^−2^ mol L^−1^ chloranilic acid, 1.0 × 10^−2^ mol L^−1^ Pb(NO_3_)_2_, and 1.0 × 10^−2^ mol L^−1^ KBrO_3_ were prepared by dissolving the reagents in water. The cupferron and chloranilic acid solutions were kept in a refrigerator prior to use; the cupferron solution was prepared once a week. The 10^−3^ mol L^−1^ stock solutions of Bi(III), Cu(II), Ni(II), Cd(II), Ca(II), Fe(III), Mg(II), and Cu(II) were prepared from Merck (Darmstadt, Germany) reagents. The influence of Triton X-100 was investigated based on a reagent obtained from Fluka (Charlotte, NC, USA).

### 2.2. Apparatus

Voltammetric studies were performed on a µAutolab electrochemical analyzer integrated with GPES 4.9 software (Eco Chemie, Utrecht, the Netherlands) and an electrode stand (M164D, MTM Anko, Krakow, Poland), in a three-electrode arrangement with a glassy carbon electrode (diameter of 1 mm) electrochemically coated with lead (GCE/PbF) as a working electrode, a platinum electrode as an auxiliary electrode, and Ag/AgCl (3 mol L^−1^ KCl) as a reference electrode. A µAutolab analyzer integrated with FRA 4.9 software was applied for electrochemical impedance spectroscopy (EIS) studies. Silicon carbide paper (SiC-paper, #4000, Buehler, Skovlunde, Denmark), alumina particle suspension (1.0, 0.3, and 0.05 µm), and a Buehler polishing pad were used to prepare the GCE surface before a series of measurements. A UV digester, made by Mineral, Poland, was used for three-hour mineralization of the certified reference material water samples (SLEW-3, estuarine water, National Research Council Canada, Ottawa, ON, Canada) and river water samples (acidified to a pH of 2.0 with nitric acid, Vistula River, Sandomierz, Poland).

### 2.3. DPAdSV Procedure Parameters

The analysis of V(V) at the GCE/PbF was carried out by differential pulse adsorptive stripping voltammetry (DPAdSV) in a solution containing 10 mL of 0.3 mol L^−1^ buffer solution (CH_3_COONH_4_, CH_3_COOH, and NH_4_Cl) of pH = 5.6 ± 0.1, 5.0 × 10^−4^ mol L^−1^ Pb(NO_3_)_2,_ and 7.0 × 10^−4^ mol L^−1^ cupferron. The DPAdSV parameters under optimized conditions of V(V) analysis at the GCE/PbF are collected in Table 1. The electrochemical cleaning step was performed at the potential of −1.1 V for 15 s and 0.2 V for 15 s. Then, lead film was deposited at the potential of −1.1 V (E_dep. Pb_) for 15 s (t_dep. Pb_), and the V(V)–cupferron complexes at −0.6 V (E_acc._) for 15 s (t_acc._) were accumulated. After the equilibrium period of 5 s, the DPAdSV curves were recorded in the potential range from −0.6 to −0.9 V. The background curve was subtracted, and the baseline was corrected for each voltammogram.

## 3. Results and Discussion

In our previous studies, we showed a simple way to amplify the analytical signal using a lead film electrode and a new supporting electrolyte composition (CH_3_COONH_4_, CH_3_COOH, and NH_4_Cl). This electrolyte was applied instead of the commonly used acetate buffer (CH_3_COONa and CH_3_COOH) in order to significantly enhanced the adsorptive stripping voltammetric signal of vanadium(V) at the lead-coated glassy carbon electrode (GCE/PbF). In order to establish the most suitable experimental conditions, the following optimization studies were performed: selection of supporting electrolyte and complexing agent, the influence of pH value of the supporting electrolyte, cupferron and Pb(II) concentrations, potential and time of lead deposition, and accumulation time of V(V)–cupferron complexes on the analytical signal V(V), as well as the effect of the DPV parameters (scan rate, amplitude, and modulation time) on the V(V) peak current.

### 3.1. Selection of Supporting Electrolyte and Complexing Agent

**Figure 1:**
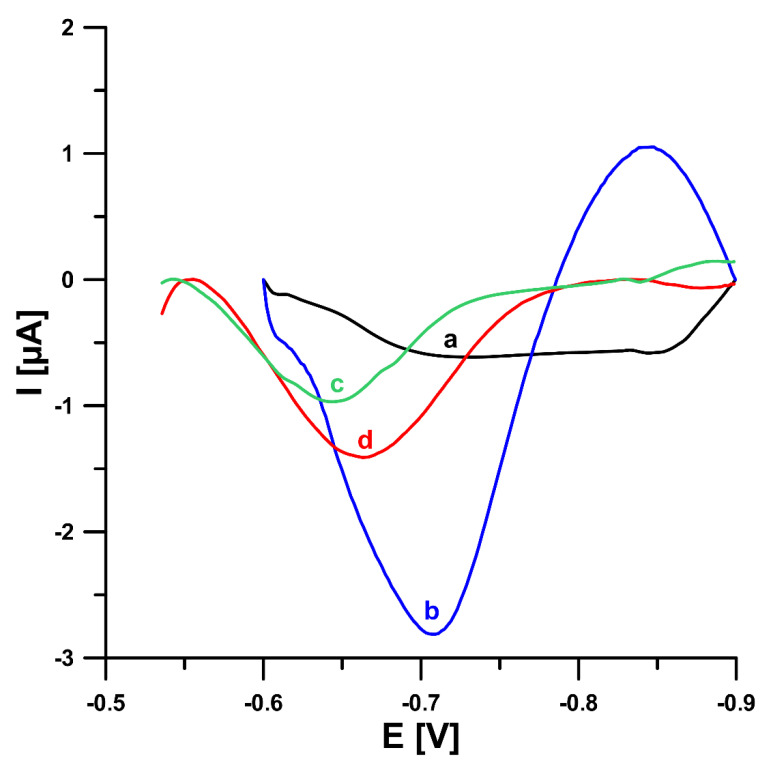
Comparison of the DPAdSV curves registered for 1.0 × 10^−8^ mol L^−1^ V(V) at the GCE/PbF in 0.3 mol L^−1^ acetate buffer solution (CH_3_COONa and CH_3_COOH) of pH 5.6 ± 0.1 (**a**,**c**) or in 0.3 mol L^−1^ buffer solution (CH_3_COONH_4_, CH_3_COOH, and NH_4_Cl) of pH 5.6 ± 0.1 (**b**,**d**) in the presence of 3.0 × 10^−4^ mol L^−1^ Pb(NO_3_)_2_ and 7.0 × 10^−4^ mol L^−1^ cupferron (**a**,**b**) or 3.0 × 10^−4^ mol L^−1^ Pb(NO_3_)_2_, 3.0 × 10^−5^ mol L^−1^ chloranilic acid and 1.0 × 10^−2^ mol L^−1^ bromate ions (**c**,**d**). The parameters: −1.1 V (E_dep. Pb_) for 20 s (t_dep. Pb_), −0.6 V (E_acc._) for 30 s (t_acc._), 100 mV (ΔE_A_), 20 mV s^−1^ (ν), and 2 ms (t_m_).

**Figure 2:**
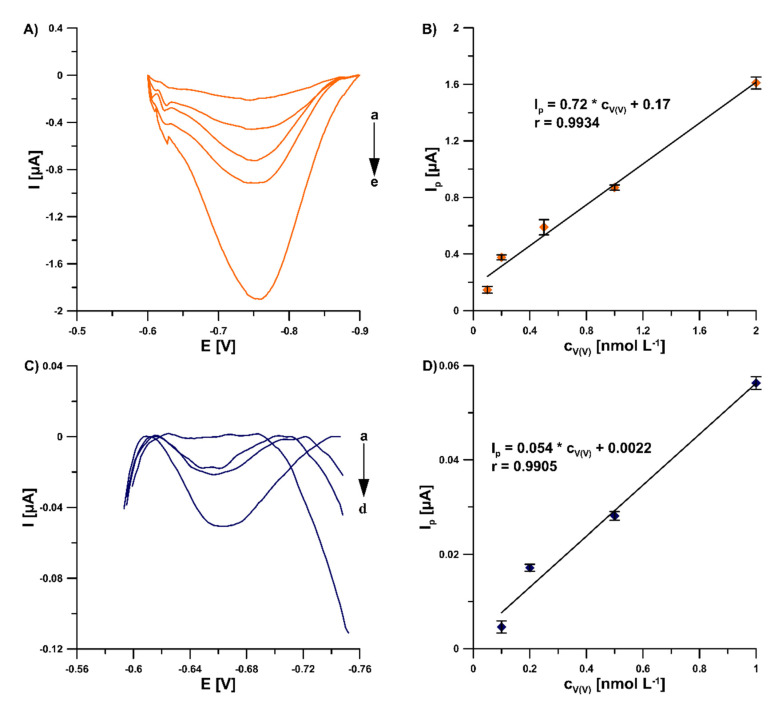
DPAdSV curves registered at the GCE/PbF in 0.3 mol L^−1^ buffer solution (CH_3_COONH_4_, CH_3_COOH, and NH_4_Cl) of pH 5.6 ± 0.1 in the presence of 3.0 × 10^−4^ mol L^−1^ Pb(NO_3_)_2_, increasing concentration of V(V) (a: 1.0 × 10^−10^, b: 2.0 × 10^−10^, c: 5.0 × 10^−10^, d: 1.0 × 10^−9^, e: 2.0 × 10^−9^ mol L^−1^) and (**A**) 7.0 × 10^−4^ mol L^−1^ cupferron, (**C**) 3.0 × 10^−5^ mol L^−1^ chloranilic acid and 1.0 × 10^−2^ mol L^−1^ bromate ions. (**B**) V(V) calibration graph in the presence of cupferron as a complexing agent, (**D**) V(V) calibration graph in the presence of chloranilic acid and bromate ions. The parameters: −1.1 V (E_dep. Pb_) for 20 s (t_dep. Pb_), −0.6 V (E_acc._) for 30 s (t_acc._), 100 mV (ΔE_A_), 20 mV s^−1^ (ν), and 2 ms (t_m_). The average values of Ip are presented with the standard deviation of n = 3.

In this study, in order to improve the V(V) analytical signal at the GCE/PbF, a new supporting electrolyte of 0.3 mol L^−1^ (CH_3_COONH_4_, CH_3_COOH and NH_4_Cl) and pH 5.6 ± 0.1 was for the first time applied, instead of a 0.3 mol L^−1^ acetate buffer solution (CH_3_COONa and CH_3_COOH) of pH 5.6 ± 0.1, in the presence of cupferron as a complexing agent [15], as well as chloranilic acid and bromate ions [8]. Figure 1 shows a comparison of the obtained DPAdSV curves. The application of the new supporting electrolyte composition is an easy way to significantly enhance the V(V) analytical signal (0.29 vs. 3.01 µA in the presence of cupferron and 0.96 vs. 1.41 µA in the presence of chloranilic acid and bromate ions). It is related to the improvement in the conductivity of the supporting electrolyte, as we wrote in an earlier work [22]. To decide which adsorptive voltammetric system to choose (in the presence of cupferron or chloranilic acid and bromate ions) for further research, measurements were performed for low concentrations of V(V) in the range of 1 × 10^−10^ to 2 × 10^−9^ mol L^−1^. Figure 2 shows the obtained DPAdSV curves and calibration graphs in the studied range of V(V) concentrations. As can be seen, the linear range of V(V) at the GCE/PbF in the presence of cupferron is wider and the sensitivity is approximately 10 times higher (from 1 × 10^−10^ to 2 × 10^−9^ mol L^−1^ with a sensitivity of 0.72 µA/nmol L^−1^) than in the presence of chloranilic acid and bromate ions (from 1 × 10^−10^ to 1 × 10^−9^ mol L^−1^ with a sensitivity of 0.054 µA/nmol L^−1^). Therefore, to develop a simple and extremely sensitive voltammetric procedure of V(V) determination using a GCE/PbF, the adsorptive voltammetric system in the presence of cupferron and a new buffer solution composition (CH_3_COONH_4_, CH_3_COOH, and NH_4_Cl) of pH 5.6 ± 0.1 were adopted.

### 3.2. Step of the Optimization Procedure

**Figure 3:**
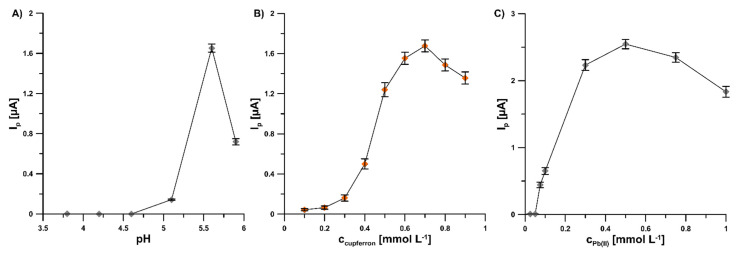
Dependence of V(V) peak current on: (**A**) pH buffer solution, cupferron (**B**), and Pb(II) (**C**) concentrations. Tested solution: 0.3 mol L^−1^ buffer solution (CH_3_COONH_4_, CH_3_COOH, and NH_4_Cl) of pH 3.5, 4.2, 4.6, 5.2, 5.6, 5.9 ± 0.1 (**A**) or 5.6 ± 0.1 (**B**,**C**), 2.0 × 10^−9^ mol L^−1^ V(V), 3.0 × 10^−4^ mol L^−1^ Pb(II) (**A**,**B**) or from 0 to 1.0 × 10^−3^ mol L^−1^ Pb(II) (**C**), and from 1.0 × 10^−4^ to 9.0 × 10^−4^ mol L^−1^ cupferron (**B**) or 7.0 × 10^−4^ mol L^−1^ cupferron (**A**,**C**). The parameters: −1.1 V (E_dep. Pb_) for 20 s (t_dep. Pb_), −0.6 V (E_acc._) for 30 s (t_acc._), 100 mV (ΔE_A_), 20 mV s^−1^ (ν), and 2 ms (t_m_). The average values of Ip are presented with the standard deviation of n = 3.

To achieve an extremely sensitive voltammetric procedure of V(V) determination at the GCE/PbF, the individual parameters were optimized. The influence of pH value of the supporting electrolyte, cupferron and Pb(II) concentrations, potential and time of lead deposition, and accumulation time of V(V)–cupferron complexes on the analytical signal V(V) was tested. The pH value of the supporting electrolyte (pH of 5.6 ± 0.1) was selected on the basis of results obtained for 2.0 × 10^−9^ mol L^−1^ V(V) (Figure 3A). The effect of the pH buffer solution over the range from 3.5 to 5.9 was investigated. The results show that an increase in the acidity of the supporting electrolyte contributes to a deterioration of the V(V) signal. It is related to the shifting of the V(V) peak towards the less negative potential values with the increase in the acidity of the solution and the overlapping of the V(V) peak on the reduction Pb(II) peak. The variation of cupferron concentration (from 1.0 × 10^−4^ to 9.0 × 10^−4^ mol L^−1^) significantly affected the peak current of 2.0 × 10^−9^ mol L^−1^ V(V) (Figure 3B). In the subsequent measurements, a cupferron concentration of 7.0 × 10^−4^ mol L^−1^ was used because the highest value of the V(V) peak current was attained. The Pb(II) concentration within the range of 0 to 1.0 × 10^−3^ mol L^−1^ significantly affected the peak current of 2.0 × 10^−9^ mol L^−1^ V(V) (Figure 3C). No V(V)–cupferron complexes were adsorbed on the unmodified electrode surface and therefore the V(V) peak was invisible. Further increases in Pb(II) concentration up to 5.0 × 10^−4^ mol L^−1^ significantly amplified the V(V) signal. Consequently, this Pb(II) concentration was applied in subsequent studies.

**Figure 4:**
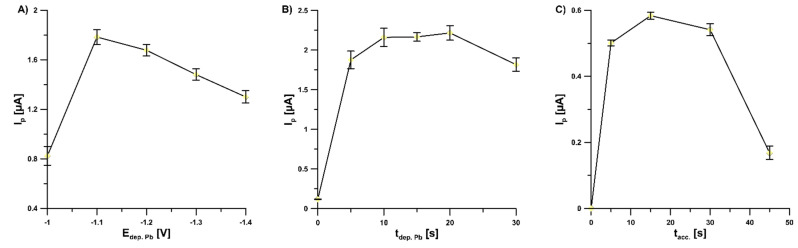
Dependence of V(V) peak current on: (**A**) E_dep. Pb_, (**B**) t_dep. Pb,_ and (**C**) t_acc_. Tested solution: 0.3 mol L^−1^ buffer solution (CH_3_COONH_4_, CH_3_COOH, and NH_4_Cl) of pH 5.6 ± 0.1, 5.0 × 10^−4^ mol L^−1^ Pb(II), 7.0 × 10^−4^ mol L^−1^ cupferron, and 1.0 × 10^−9^ mol L^−1^ V(V) (**A**,**B**) or 5.0 × 10^−10^ mol L^−1^ V(V) (**C**). The parameters: 100 mV (ΔE_A_), 20 mV s^−1^ (ν), and 2 ms (t_m_). The average values of Ip are presented with the standard deviation of n = 3.

The investigation of the effect of Pb deposition potential (E_dep. Pb_, from −1.1 to −1.4 V) on the values of 2.0 × 10^−9^ mol L^−1^ V(V) peak current showed that the highest response was achieved at a potential of −1.1 V and therefore this value of E_dep. Pb_ was selected for further studies (Figure 4A). Furthermore, the influence of Pb deposition time (t_dep. Pb_, from 0 to 30 s) on the values of 2.0 × 10^−9^ mol L^−1^ V(V) peak current was studied (Figure 4B). The highest V(V) responses were obtained within t_dep. Pb_ in the range of 10–20 s; the value of 15 s was chosen for further studies.

Accumulation potential and time are always important factors since they affect the linear range of the calibration graph and detection limit of the voltammetric procedure. The optimal potential of accumulation (E_acc_.) of V(V)–cupferron complexes onto the GCE/PbF surface in the buffer solution (CH_3_COONH_4_, CH_3_COOH, and NH_4_Cl) of pH 5.6 ± 0.1 was −0.6 V. For the E_acc_. lower than −0.6 V and higher than −0.6 V, the V(V) signal decreased significantly. It is connected with a narrow range of available potentials between the Pb(II) and V(V) reduction signals. Additionally, the impact of accumulation time of V(V)–cupferron complexes (t_acc..,_ from 0 to 45 s) on the peak currents of 5.0 × 10^−10^ mol L^−1^ V(V) was investigated (Figure 4C). As can be seen, the highest V(V) peak current was achieved for 15 s and hence this value was chosen as optimal.

The effect of the DPV parameters (scan rate (ν), amplitude (ΔE_A_), and modulation time (t_m_)) on the 2.0 × 10^−9^ mol L^−1^ V(V) peak current was examined. For t_m_ of 2 ms and ν of 20 mV s^−1^, the ΔE_A_ was changed from 25 to 125 mV. The best results were obtained for the ΔE_A_ of 100 mV. The ΔE_A_ higher than 100 mV caused a major increase in the background current. Next, the dependence of the ν values, ranging from 10–100 mV s^−1^, on the 2.0 × 10^−9^ mol L^−1^ V(V) signal was studied. The highest V(V) signal was found at the ν value of 20 mV s^−1^, so this value was used for subsequent experiments. Furthermore, the t_m_ was varied from 2 to 10 ms. For the t_m_ of 2 ms, the highest 2.0 × 10^−9^ mol L^−1^ V(V) peak current was achieved and therefore this value was selected as optimal.

### 3.3. Electrochemical Characteristics of the Sensor

**Figure 5:**
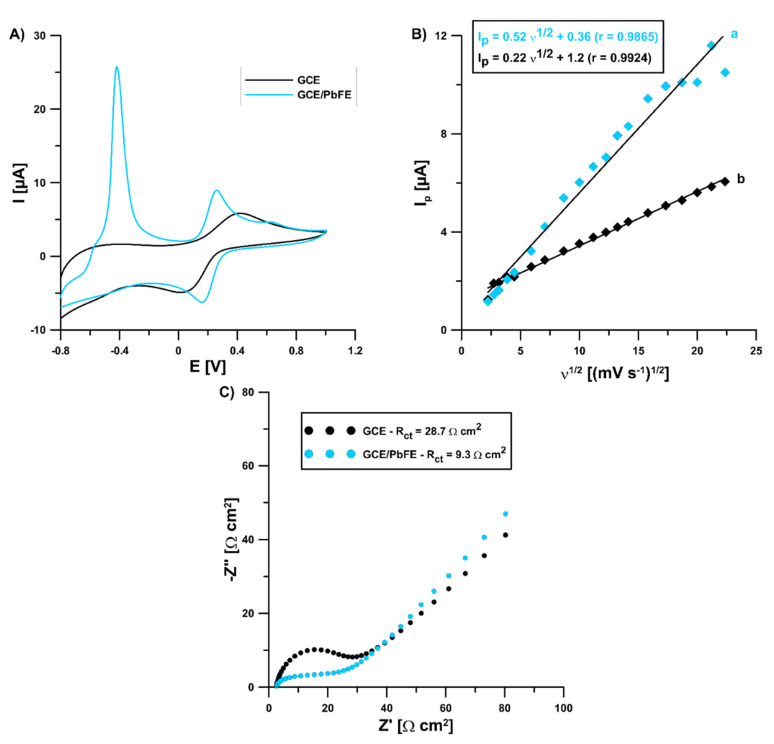
(**A**) CVs obtained at a bare GCE and GCE/PbF using the υ of 100 mV s^−1^; (**B**) the dependence between the anodic peak current (I_p_) and the square root of the υ (υ^1/2^) at the GCE (b) and GCE/PbF (a); (**C**) Nyquist plots of the GCE and GCE/PbF.

In the solution of 0.1 mol L^−1^ KCl containing 5 × 10^−3^ mol L^−1^ K_3_[Fe(CN)_6_] and 0 (for GCE) and 3.0 × 10^−4^ mol L^−1^ Pb(II) (for GCE/PbF), electrochemical characteristics were investigated for the lead-coated and bare glassy carbon electrode using cyclic voltammetry (CV) and electrochemical impedance spectroscopy (EIS). The CV curves were registered in the υ range of 5.0–500 mV s^−1^ and they showed a pair of well-shaped redox peaks of (Fe(CN)_6_)^3−/4−^ at the bare GCE and GCE/PbF (Figure 5A for υ of 100 mV s^−1^). However, the signal intensity at the modified electrode was much better (anodic: 3.52 vs. 5.95 µA, cathodic: 3.88 vs. 5.49 µA, respectively). Moreover, in the case of the GCE/PbF a new peak appeared at a potential of −0.42 V, which is related to the oxidation of the deposited lead. Covering electrochemically the electrode surface with lead causes an acceleration of electron transfer kinetics and contributes to increasing the active surface of the electrode. The relative peak separation (χ°) was equal to 1.66 for the GCE/PbF and 5.97 for the GCE. The χ° value for the GCE/PbF was closer to the theoretical value of 1.0. On the other hand, the electrochemically active electrode area (A_s_) of the GCE and GCE/PbF was calculated to be 0.19 and 0.46 mm^2^, respectively (Figure 5B). Moreover, the GCE/PbF is characterized by a lower charge transfer resistance (R_ct_) (28.7 vs. 9.3 Ω) and good conductivity, which was found on the basis of the EIS measurements (Figure 5C). The Nyquist plots were registered at a potential of 0.2 V and in a frequency range from 50 kHz to 1 Hz.

### 3.4. Calibration Graph, Repeatability, and Reproducibility

**Figure 6:**
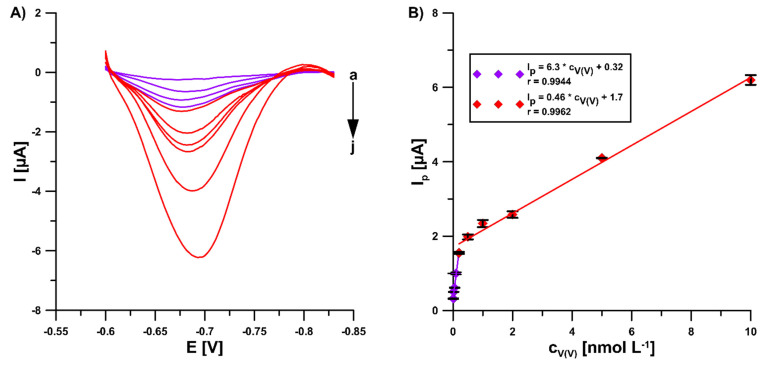
(**A**) The DPAdSV curves recorded in 0.3 mol L^−1^ buffer solution (CH_3_COONH_4_, CH_3_COOH, and NH_4_Cl) of pH 5.6 ± 0.1, 5.0 × 10^−4^ mol L^−1^ Pb(II), 7.0 × 10^−4^ mol L^−1^ cupferron at the GCE/PbF in the presence of various V(V) concentrations (a → j, 1.0 × 10^−11^–2.0 × 10^−8^ mol L^−1^). (**B**) V(V) calibration graph. The parameters: 100 mV (ΔE_A_), 20 mV s^−1^ (ν), and 2 ms (t_m_). The average values of Ip are presented with the standard deviation of n = 3.

In the optimal conditions (0.3 mol L^−1^ buffer solution (CH_3_COONH_4_, CH_3_COOH, and NH_4_Cl) of pH 5.6 ± 0.1, 5.0 × 10^−4^ mol L^−1^ Pb(II), 7.0 × 10^−4^ mol L^−1^ cupferron, −1.1 V (E_dep. Pb_) for 15 s (t_dep. Pb_), −0.6 V (E_acc._) for 15 s (t_acc._), 100 mV (ΔE_A_), 20 mV s^−1^ (ν), and 2 ms (t_m_)), the DPAdSV peak current increased linearly with the V(V) concentration from 1.0 × 10^−11^ to 2.0 × 10^−10^ mol L^−1^ and 2.0 × 10^−10^ to 1.0 × 10^−8^ mol L^−1^. The DPAdSV curves and the V(V) calibration graph are presented in Figure 6. The detection (LOD) and quantification (LOQ) limits were estimated to be 2.8 × 10^−12^ and 9.3 × 10^−12^ mol L^−1^, respectively, using the LOD = 3SD_a_/b and LOQ = 10 SD_a_/b equations (SD_a_—standard deviation of intercept (n = 3); b—slope of calibration curve) [25]. The peak current standard deviation values for all concentrations of V(V) from the calibration graph in the range of 0.14–4.2 % (n = 3) confirmed satisfactory signal repeatability. Moreover, three GCE/PbF were prepared independently and used for the determination of 2.0 × 10^−9^ mol L^−1^ V(V). The RSD of 5.5% (n = 9) confirms the acceptable reproducibility of a new sensor.

Table 2 shows comparison of voltammetric procedures for the analysis of V(V). It can be seen that the voltammetric procedure described in this article offers the lowest detection limit for the lowest accumulation time.

### 3.5. Selectivity Studies

To check the proposed procedure selectivity, the 2.0 × 10^−9^ mol L^−1^ V(V) signals were registered in the presence of potential interferents. It was found that a 1000-fold excess of Ca(II), Mg(II), Bi(III), and Cu(II) as well as a 100-fold excess of Ni(II), Fe(III), and Cd(II) did not change the peak current of V(V) by more than 5%. According to the literature data [26], natural waters contain surfactants with a surface-active effect corresponding to 0.2–2.0 ppm of Triton X-100; therefore, the effect of the Triton X-100 presence in the solution on the 2.0 × 10^−9^ mol L^−1^ V(V) signal was studied. It was found that 0.5 ppm of Triton X-100 caused a suppression of the V(V) signal to 90% of its original value. The presence of organic sample components that can block the electrode surface is a major limitation of the practical application of voltammetric techniques. In the case of the proposed procedure, this problem was solved by the decomposition of the organic matrix during UV mineralization of the samples.

### 3.6. Samples Analysis

The practical application of the elaborated DPAdSV procedure was evaluated by quantification of the total V in UV-mineralized samples of certified reference material (SLEW-3, estuarine water) and Vistula River water samples using the standard addition method. The voltammetric results are summarised in Table 3. No significant difference was found between the determined concentration and the certified quantity value (the relative error of 1.8%). As can be seen, V(V) was determined in the Vistula River samples at a concentration of 4.7 × 10^−9^ mol L^−1^. The recovery values were between 98.6 and 105.2%, which confirms a satisfactory degree of accuracy of the procedure.

## 4. Conclusions

The article presents a simple, sensitive, and selective voltammetric procedure for the determination of V(V) using a new supporting electrolyte composition (CH_3_COONH_4_, CH_3_COOH, and NH_4_Cl) and the lead-coated glassy carbon electrode (GCE/PbF) as a working electrode. The results showed that the application of a new buffer solution (CH_3_COONH_4_, CH_3_COOH, and NH_4_Cl), instead of the commonly used acetate buffer (CH_3_COONa and CH_3_COOH), contributed to a high increase in the V(V) signal at the GCE/PbF in the presence of cupferron as a complexing agent as well as by using the catalytic system of V-chloranilic acid–bromate ions. However, the highest sensitivity can be obtained by using the new supporting electrolyte, the GCE/PbF sensor, and cupferron as a complexing agent. Such a simple electrolyte change allows us to obtain the lowest detection limit of V(V) (2.8 × 10^−12^ mol L^−1^ for an accumulation time of 15 s) in comparison with the previously described voltammetric procedures at mercury and less toxic electrodes. It is worth emphasizing that lead and lead salts are toxic but less toxic and less volatile than mercury and mercury salt used for the preparation of mercury electrodes. Therefore, the application of the proposed procedure of V(V) determination at the GCE/PbF sensor in the new supporting electrolyte allows us to eliminate mercury and mercury-salt waste. This study represents an additional step towards the replacement of mercury electrodes in adsorptive stripping voltammetric analysis of metal ions. The attractive behaviour of V(V) in a new supporting electrolyte composition indicates great promise for developing SV procedures of other metal ions and biologically active compound analysis in a manner similar to that reported for mercury electrodes.

## Figures and Tables

**Table 1:** The DPAdSV parameters under optimized conditions of the V(V) analysis at the GCE/PbF.

Procedure Step	Parameters
Electrochemical cleaning step Lead film deposition	−1.1 V for 15 s and 0.2 V for 15 s −1.1 V (E_dep. Pb_) for 15 s (t_dep. Pb_)
V(V)–cupferron accumulation	−0.6 V (E_acc._) for 15 s (t_acc._)
Signal registration	from −0.6 to −0.9 V
	scan rate of 20 mV s^−1^ (ν)
	amplitude of 100 mV (ΔE_A_) modulation time of 2 ms (t_m_)

**Table 2:** Comparison of voltammetric procedures for V(V) analysis.

Electrode	Complexing Agent	Linear Range [mol L^−1^] /Accumulation Time [s]	LOD [mol L^−1^]	Application	Ref.
HMDE	chloranilic acid	4.9 × 10^−8^–2.9 × 10^−7^/15	2.7 × 10^−9^	CRM, see, local portable water, sewage sample	[11]
BiFE	chloranilic acid	9.8 × 10^−8^–5.0 × 10^−7^/600	3.9 × 10^−9^	groundwater	[12]
HMDE	chloranilic acid	2.0 × 10^−10^–5.0 × 10^−8^/100	9.0 × 10^−12^	CRM	[13]
Hg(Ag)FE	chloranilic acid	2.5 × 10^−10^–1.0 × 10^−7^/90	1.0 × 10^−11^	CRM, tap water	[14]
PbFE	cupferron	1.0 × 10^−9^–7.0 × 10^−8^/30	3.2 × 10^−10^	CRM, river water	[15]
BiFµE	cupferron	8.0 × 10^−10^–1.0 × 10^−7^/60	2.5 × 10^−10^	CRM, river, rain, and tap water	[16]
MWE	gallic acid	1.0 × 10^−10^–2.0 × 10^−8^/120	1.7 × 10^−11^	river water	[17]
Polystyrene-coated BiFE	gallic acid	2.0 × 10^−9^–2.0 × 10^−8^/600	-	-	[17]
CPE	alizarin red S	2.0 × 10^−9^–3.0 × 10^−7^/120	7.8 × 10^−10^	tap, river, and groundwater	[18]
ABPE	alizarin violet	8.0 × 10^−10^–1.0 × 10^−7^/90	6.0 × 10^−10^	tap, river, and mineral water	[19]
SME	2,3-dihydroxybenzaldehide	5.0 × 10^−10^–5.0 × 10^−8^ / 30	6.0 × 10^−10^	river water	[20]
HMDE	QSA	No data–7.0 × 10^−9^/30	4.5 × 10^−12^	tap, purified drinking, river, and commercial water for chromatography	[21]
GCE/PbF	cupperon	1.0 × 10^−11^–2.0 × 10^−10^/15 2.0 × 10^−10^–2.0 × 10^−8^	2.8 × 10^−12^	CRM river water	This work

CRM—certified reference material, HMDE—hanging mercury drop electrode, BiFE—bismuth film electrode, Hg(Ag)FE—renewable mercury film silver-based electrode, BiFµE—solid bismuth microelectrode, MWE—mercury-coated gold micowire electrode, CPE—carbon paste electrode, ABPE—acetylene black paste electrode, SME—stationary mercury electrode, QSA—quercetin-5-sulfonic acid, ND.

**Table 3:** Results of V(V) determination in environmental water samples.

**Sample**	**Measured Value ± SD (n = 3) [mol L^−1^]**	**Certified Value ± SD (n = 3) [mol L^−1^]**	**Recovery [%]**	**Relative Error [%]**
CRM				
(estuarine water)	4.96 × 10^−8^ ± 0.51	5.05 × 10^−8^ ± 0.609	98.2	1.8
**Sample**	**Added [mol L^−1^]**	**Found** **± SD (n = 3) [mol L^−1^]**	**Recovery [%]**	**Relative Error [%]**
Vistula	0	4.7 × 10^−9^ ± 0.24	-	-
River	5.0 × 10^−9^	10.2 × 10^−9^ ± 0.52	105.2	5.2
	10.0 × 10^−9^	14.5 × 10^−9^ ± 0.69	98.6	1.4

## Data Availability

The data presented in this study are available on request from the corresponding author.
